# Serum levels of presepsin reffects the APACHE II and SOFA scores in patients with sepsis

**DOI:** 10.1186/cc11975

**Published:** 2013-03-19

**Authors:** R Sato, Y Suzuki, M Sato, G Takahashi, M Kojika, Y Inoue, S Endo

**Affiliations:** 1 Iwate Medical University, Morioka, Japan

## Introduction

As a method for the diagnosis of sepsis, we previously reported an ELISA method for measuring the serum levels of presepsin. That method, however, took approximately 2 hours to yield results.

## Methods

To resolve this problem, we later developed a simplified assay kit making use of immunochromatography (Point of Care test; POC test), and are currently evaluating the usefulness of this kit for diagnosis of sepsis and evaluation of its severity. In 21 septic patients with sepsis, we analyzed the serum levels of presepsin in relation to APACHE II and SOFA scores.

## Results

APACHE II and SOFA scores at baseline were 28.7 ± 7.5 and 10.8 ± 3.9, respectively, in these patients. These were significant correlations between the serum presepsin levels and the APACHE II score, and also between the serum presepsin levels and the SOFA score.

## Conclusion

Furthermore, there was also a significant correlation between the results of the POC test and the serum presepsin levels. These results indicate that measurement of the serum presepsin might be useful for evaluating the severity of sepsis.

